# A Multi-Modal Remote Sensing Image Collaborative Fusion Network for Construction and Demolition Waste Extraction in Urban Areas

**DOI:** 10.3390/s26144618

**Published:** 2026-07-21

**Authors:** Ya-Zhe Xie, Zhong-Qi Shi, Lan-Qing Zhang, Yu-Zhou Liu, Nan-Hua-Nuo-Wa Zhu, Hong Zhang

**Affiliations:** 1 Key Laboratory of Digital Earth Science, Aerospace Information Research Institute, Chinese Academy of Sciences, Beijing 100094, China; xieyazhe24@mails.ucas.ac.cn; 2 International Research Center of Big Data for Sustainable Development Goals, Beijing 100094, China; 3 University of Chinese Academy of Sciences, Beijing 100049, China; 4 China Shenzhen Technology Institute of Urban Public Safety, Shenzhen 518000, China; yuzhouliu@link.cuhk.edu.hk; 5 Urban Safety Development Institute of Science and Technology (Shenzhen), Shenzhen 518023, China; 6 China Spacesat Co., Ltd., Beijing 100081, China; zhanglanqing2006@126.com; 7 Satellite Application Center for Ecology and Environment, Ministry of Ecology and Environment, Beijing 100094, China; zhunanhuanuowa@163.com; 8 Key Laboratory of Satellite Remote Sensing, Ministry of Ecology and Environment, Beijing 100094, China

**Keywords:** construction and demolition waste (C&DW), remote sensing, deep learning, multimodal semantic segmentation

## Abstract

**Highlights:**

**What are the main findings?**

- The first optical-SAR multimodal dataset for fine-scale C&DW segmentation and a dual-branch CNN–Mamba–Transformer fusion framework.
- Cross-modal frequency interaction for optical-SAR complementary enhancement and multi-source boundary attention for fragmented and irregular C&DW delineation.

**What are the implications of the main findings?**

- Shift from single-modality C&DW monitoring to optical-SAR collaborative perception in complex urban environments.
- Boundary-aware multi-scale fusion significantly improves target delineation in highly confused urban backgrounds.

**Abstract:**

Construction and demolition waste (C&DW) mapping is crucial for urban environmental management, yet accurate extraction remains challenging due to complex backgrounds, spectral confusion, and irregular boundaries. To address these issues, this study presents the first optical-SAR multimodal semantic segmentation dataset based on GF-3 SAR, Sentinel-1 SAR, and Sentinel-2 optical imagery with a unified spatial resolution of 3 m for fine-scale C&DW extraction and proposes CDW-Net, a dual-branch multimodal fusion framework. The framework incorporates a Cross-Frequency Interaction (CFI) module for cross-modal feature fusion and a Multi-Source Boundary Attention Module (MS-BAM) for improved boundary representation. Experiments in Beijing show that CDW-Net achieves an IoU of 0.8470 and an F1-score of 0.9171, outperforming state-of-the-art unimodal and multimodal methods. Transfer experiments in Guangzhou achieved an IoU of 0.8247 and an F1-score of 0.9040 on manually interpreted validation samples, demonstrating good cross-regional transferability. The proposed dataset and framework provide valuable support for fine-scale C&DW monitoring in complex urban environments.

## 1. Introduction

Driven by continuous economic development and accelerated urban-rural modernization, the scale of construction and related industries has expanded significantly, leading to a steady increase in the volume of construction and demolition waste (C&DW) [1,2,3]. This has emerged as a major environmental and economic challenge [4,5,6]. Excessive waste generation not only strains landfill capacity and resource recovery but also triggers issues such as illegal dumping, improper disposal, and greenhouse gas emissions [7,8,9]. Consequently, the timely and accurate acquisition of information regarding the spatial distribution, accumulation extent, and area of C&DW is of great significance for urban environmental supervision and waste management.

Compared with traditional manual field surveys, remote sensing technology offers distinct advantages, including extensive spatial coverage, high acquisition efficiency, and abundant spatial information, providing an effective means for the automated identification and fine-grained extraction of C&DW [10,11,12]. High-quality datasets serve as the essential foundation for the training, performance evaluation, and practical deployment of intelligent remote sensing interpretation models. However, existing C&DW-related remote sensing datasets, such as the AerialWaste [13] and DSWD [14], have mainly focused on scene classification or object detection tasks, making them insufficient for the pixel-level extraction of boundary and area information [15]. Datasets for semantic segmentation, such as CWLD [16], still rely predominantly on optical imagery, which mainly reflects the spectral, textural, and spatial morphological characteristics of the targets. In complex urban environments, C&DW is characterized by complex material compositions, fragmented morphologies, and irregular boundaries, which are easily confused with land cover types such as bare soil, construction sites, and stockpiles. Synthetic Aperture Radar (SAR) data can provide complementary information related to surface structure, roughness, and backscattering characteristics. Coupled with its all-day and all-weather observation capabilities, SAR helps enrich the feature representation of C&DW [17]. Therefore, the construction of a C&DW semantic segmentation dataset that integrates both optical and SAR information can provide more robust data support for fine-grained identification in complex urban environments.

Existing C&DW identification methods can be categorized into machine learning and deep learning approaches. Machine learning-based methods enable rapid C&DW identification; for example, a 2021 study compared three classification algorithms—CART decision trees, Random Forest (RF), and Support Vector Machines (SVM)—with RF achieving the optimal classification accuracy. In recent years, deep learning methods have advanced rapidly in C&DW identification and fine-grained extraction due to their end-to-end feature learning capabilities [18]. Previous studies have applied semantic segmentation networks such as U-Net, DeepLabV3+, PSPNet, and HRNet to high-resolution optical remote sensing imagery (e.g., GF-2, ZY-3). U-Net effectively preserves fine spatial details through encoder-decoder skip connections, DeepLabV3+ enlarges the receptive field using atrous convolutions, PSPNet aggregates multi-scale contextual information through pyramid pooling, and HRNet maintains high-resolution feature representations throughout the network. These architectures have demonstrated strong performance in optical C&DW semantic segmentation [19,20,21,22]. Furthermore, some researchers have improved classical deep learning networks to address characteristics such as significant scale variations, irregular morphologies, and complex backgrounds inherent to C&DW. For instance, CCMGNet improves multi-band feature representation through dual-branch encoding and gated fusion [23]. In 2024, a study introduced the CBAM attention module into U-Net to enhance the representation of key features for construction and demolition waste (C&DW) [24]. Another study in the same year utilized deep network architectures such as ResNet-101 for C&DW site extraction [25]. Recently, transformer-based and state space models have shown remarkable potential in remote sensing image interpretation by improving long-range dependency modeling. Vision Transformers capture global contextual information through self-attention mechanisms, whereas Mamba-based architectures achieve efficient long-range feature modeling with linear computational complexity. These architectures have demonstrated promising performance in remote sensing semantic segmentation and multimodal feature learning, and have recently shown increasing potential for optical-SAR feature fusion [26,27,28].

Despite these advances, most existing C&DW extraction methods are designed for single-source optical data, and extending them to multi-source optical and SAR scenarios presents significant challenges. Recent optical-SAR segmentation methods, such as ASANet [29], MCANet [30], and PAD [31], have explored dual-branch feature extraction and cross-modal fusion. However, these methods still face challenges in handling the heterogeneous characteristics of optical and SAR data and preserving fine boundary details for irregular C&DW targets. First, optical imagery relies primarily on spectral and textural information, whereas SAR imagery characterizes surface structural features based on microwave scattering mechanisms. These distinct imaging principles and feature representations result in pronounced cross-modal heterogeneity. Consequently, employing a unified backbone for feature extraction fails to simultaneously account for both optical detail representation and SAR structural modeling, thereby limiting the effective characterization of multi-source information. Second, when existing C&DW identification methods are extended to multi-source data through simple techniques such as feature concatenation, they lack a targeted modeling mechanism to address the discrepancies between optical and SAR features. Such fusion approaches often overlook the complementary relationship between the detailed information of the optical modality and the structural information of the SAR modality. As a result, the fused features remain insufficiently discriminative for separating C&DW from spectrally similar land cover types. Furthermore, C&DW targets are inherently characterized by irregular morphologies and fragmented boundaries. Although SAR imagery provides valuable structural information for distinguishing C&DW from spectrally similar land cover types, its inherent speckle noise and scattering effects may also introduce boundary discontinuities and feature degradation during multimodal feature interaction.

To address these issues, this study constructs the first Optical-SAR multimodal semantic segmentation dataset for fine-grained C&DW extraction based on GF-3 SAR, Sentinel-1 SAR, and Sentinel-2 optical imagery. This combination integrates high-resolution SAR structural information with complementary optical spectral characteristics, providing a robust data foundation for distinguishing C&DW from spectrally similar urban land cover types. Second, a dual-branch fusion architecture, CDW-Net, based on CNN-Mamba heterogeneous encoding and Transformer decoding, is proposed for fine-grained C&DW extraction. This heterogeneous design exploits the complementary strengths of InternImage for local optical texture representation and VMamba for efficient long-range SAR structural modeling. On this basis, a cross-frequency fusion and boundary enhancement mechanism is designed for multi-source remote sensing feature interaction. Specifically, the Cross-Frequency Interaction (CFI) module is proposed to achieve complementary modeling of optical and SAR data at the level of detailed and structural information through frequency decomposition and cross-modal bidirectional guidance. Furthermore, the Multi-Source Boundary Attention Module (MS-BAM) is designed to enhance the representation capability of irregular C&DW boundaries through multi-scale boundary modeling and local–global consistency constraints.

## 2. Study Area and Data

### 2.1. Study Area

**Figure 1:**
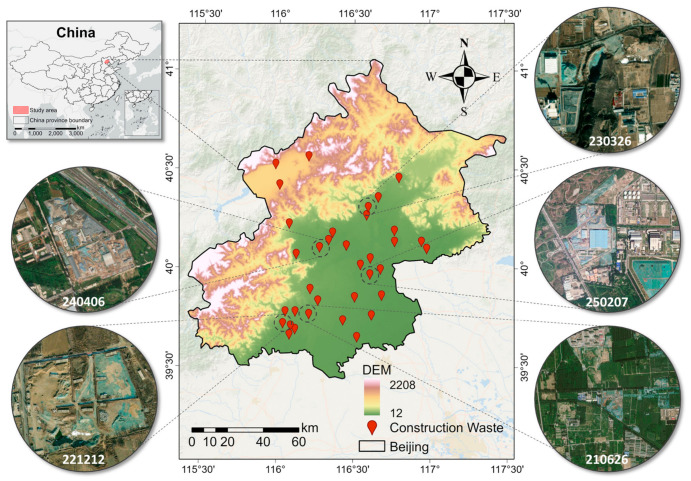
Overview of the study area and locations of C&DW sites.

Beijing, located in the northern North China Plain, is a representative megacity in China (Figure 1). The study area exhibits a transition in topography from mountainous regions in the northwest to plains in the southeast, resulting in complex land cover patterns shaped by intensive urban development and diverse landscapes. It includes various land cover types, such as high-density built-up areas, urban-rural transitional zones, industrial land, and croplands, demonstrating significant spatial heterogeneity. In recent years, continuous urban renewal and construction activities have led to the widespread distribution of C&DW across demolition sites, construction sites, and suburban areas in Beijing. These sites vary considerably in scale and exhibit fragmented and dispersed spatial patterns. In addition, owing to the composition of concrete, bricks, and waste soil, C&DW often exhibits high spectral similarity to surrounding land cover types, such as bare land and construction sites. Moreover, the generation and removal of C&DW are closely associated with construction cycles, resulting in strong temporal dynamics. In this study, 35 representative C&DW sites were selected across Beijing, covering diverse urban construction backgrounds and surface environments.

### 2.2. Data Selection and Preprocessing

This study collected multi-source remote sensing imagery covering C&DW sites in Beijing from 2021 to 2025, including GF-3 SAR, Sentinel-1 SAR, and Sentinel-2 optical data. Because the three sensors differ in acquisition time, spatial coverage, and image quality, a temporal matching strategy was adopted during data selection. Specifically, GF-3 acquisition dates were used as the temporal reference, and the nearest available Sentinel-1 and Sentinel-2 observations were selected to construct multi-source image pairs. Samples without suitable temporal matches were discarded. The temporal difference between GF-3 and the matched Sentinel observations was generally limited to within one revisit cycle, with a maximum temporal baseline of 12 days, to ensure temporal consistency among the multi-source imagery and the corresponding pixel-level labels. Meanwhile, imagery was also selected to ensure sufficient spatial coverage, image quality, and target discernibility.

The GF-3 dataset consists of 53 Level-1A scenes acquired in Ultra-Fine Stripmap (UFS) mode with HH polarization and a spatial resolution of approximately 3 m. The Sentinel-1 dataset includes 48 Ground Range Detected (GRD) scenes acquired in Interferometric Wide Swath (IW) mode with VH polarization at 10 m resolution. The Sentinel-2 dataset contains 80 Surface Reflectance (SR) Harmonized scenes, from which bands B2, B3, and B4 were used to generate 10 m RGB imagery. These bands were selected as the optical input to emphasize the complementary feature representation between optical imagery and SAR data within the proposed multimodal framework. A coefficient-of-variation feature derived from the GF-3 HH-polarized backscatter ($CV_{HH}$) was also constructed to characterize local scattering heterogeneity. The SAR datasets mainly provided backscattering and structural information, while Sentinel-2 imagery supported target interpretation, sample labeling, and optical feature extraction.

Each data source was preprocessed independently. For the GF-3 UFS data, preprocessing included radiometric calibration, multi-looking, Lee filtering, and geocoding. The Sentinel-1 GRD data were acquired through the Google Earth Engine (GEE) platform and underwent the standard preprocessing procedures provided by GEE, including orbit file application, thermal noise removal, radiometric calibration, and terrain correction. Subsequently, additional preprocessing was performed following the Sentinel-1 Analysis Ready Data (ARD) framework proposed by Mullissa et al. [32], including border noise removal, speckle filtering, and radiometric terrain normalization. The Sentinel-2 SR Harmonized data were also obtained via the GEE platform, with cloud masking performed using the Cloud Score+ dataset. Finally, geometric co-registration and bilinear resampling were performed in ENVI 5.6 to unify all imagery into the same spatial reference system and spatial resolution (3 m), thereby ensuring pixel-level spatial alignment among the multi-source imagery for subsequent model training.

## 3. Methodology

**Figure 2:**
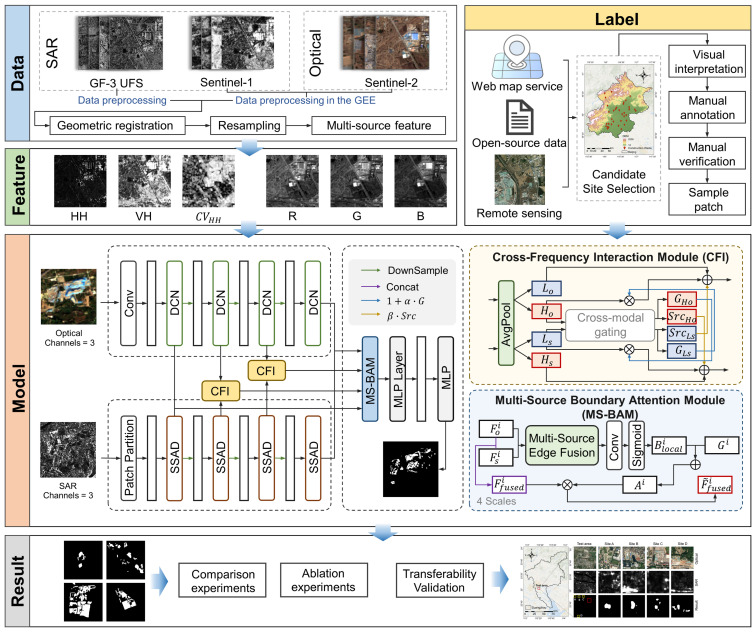
Overall research framework.

The research workflow of this study is illustrated in Figure 2. First, based on available data, a high-precision optical-SAR multi-source dataset was constructed via manual visual interpretation. Second, we propose CDW-Net, a dual-branch fusion framework for fine-grained C&DW extraction in complex urban environments. To effectively fuse heterogeneous optical and SAR features, a cross-frequency interaction mechanism was introduced to achieve complementary modeling of detailed optical information and SAR structural features. In addition, a multi-source boundary attention mechanism was developed to address the fragmented boundaries and irregular morphologies of C&DW. By integrating multi-scale boundary priors, the proposed mechanism enhances boundary awareness and improves the extraction accuracy and boundary integrity of C&DW in complex backgrounds.

### 3.1. Dataset Construction

#### 3.1.1. Label Construction

The construction of the C&DW dataset is based on candidate accumulation sites as the basic spatial units. First, in conjunction with public records, map information, and preliminary remote sensing imagery interpretation, typical C&DW accumulation sites within the study area were identified and designated as candidate regions for subsequent dataset construction. On this basis, the corresponding imagery coverage around each candidate site was extracted to form the sample areas to be labeled.

**Figure 3:**
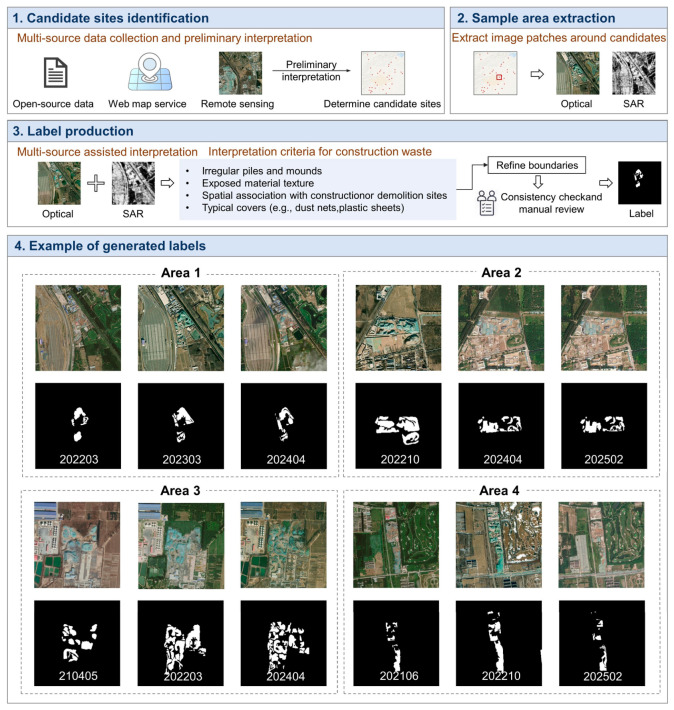
Workflow and examples of dataset preparation.

The labels were generated through manual visual interpretation. During the annotation process, optical imagery served as the primary basis for interpretation, while structural and scattering discrepancies in SAR imagery were incorporated to finely delineate C&DW boundaries. The interpretation mainly relied on irregular accumulation morphology, exposed material texture, spatial association with construction or demolition sites, and typical coverage characteristics such as dust-proof nets and tarpaulins [33]. For easily confused targets, such as bare land, construction sites, hardened surfaces, and material stockyards, comprehensive differentiation was performed by integrating target morphology, texture continuity, surrounding environment, and multi-source imagery performance. To minimize annotation uncertainty, consistency checks and manual review were conducted, while target regions that could not be reliably confirmed were excluded from valid C&DW labels. This process ultimately yielded binary C&DW labels, where C&DW regions were assigned a value of 1 and non-C&DW regions a value of 0 (Figure 3). Overall, the dataset comprises 35 representative C&DW accumulation sites, covering a total geographic area of approximately 85.11 km^2^, with multi-temporal remote sensing observations available for each site.

#### 3.1.2. Feature Construction for C&DW Identification

To fully characterize the scattering properties and spectral features of C&DW and its surrounding land cover types, this study constructs a multi-source feature system integrating SAR and optical remote sensing information to serve as the input for the subsequent classification model (Table 1).

The SAR feature group consists of three bands: HH polarization backscattering intensity, VH polarization backscattering intensity, and the HH polarization coefficient of variation (CV). The HH and VH polarization backscattering intensities are directly acquired from the SAR imagery and converted to the linear power domain. To further characterize the spatial heterogeneity of surface scattering, the CV feature is constructed based on the unresampled HH polarization channel. The CV describes the relative fluctuation degree of backscattering intensity within a local neighborhood and is highly sensitive to texture variations. It can effectively distinguish homogeneous surfaces, such as water bodies and bare land, from heterogeneous surfaces with strong texture variations, such as C&DW and vegetation. It is defined as follows:

(1) $$CV_{HH}=\frac{\sigma_{local}}{\mu_{local}}$$

where $\mu_{local}$ and $\sigma_{local}$ represent the local mean and standard deviation of the linear backscattering values within a 3 × 3-pixel sliding window centered on the target pixel, respectively. Finally, the three bands of HH, VH, and ${\mathrm{CV}}_{HH}$ are stacked to generate a three-band SAR feature image.

The optical feature group utilizes the R, G, and B bands, which are combined to form a standard true-color feature image. The visible bands reflect the spectral reflectance characteristics of different land cover types, providing a complementary contrast to the surface structural and scattering information characterized by the SAR data. By integrating these optical and SAR features, the model’s capability to identify complex artificial features, such as C&DW landfills, can be significantly enhanced.

Finally, the feature imagery and label maps were synchronously cropped to a uniform dimension to form spatially corresponding paired samples. To prevent spatial leakage during data partitioning, the samples were split based on individual accumulation sites or regions rather than a simple random partitioning of image patches, thereby ensuring relatively independent spatial distributions between the training and test sets. The image patch size was set to 256×256 pixels with an overlap rate of 0.5, ultimately yielding a total of 5898 sample pairs. These samples were partitioned into the training set and test set at an 8:2 ratio.

### 3.2. Construction of the C&DW Identification Model

**Figure 4:**
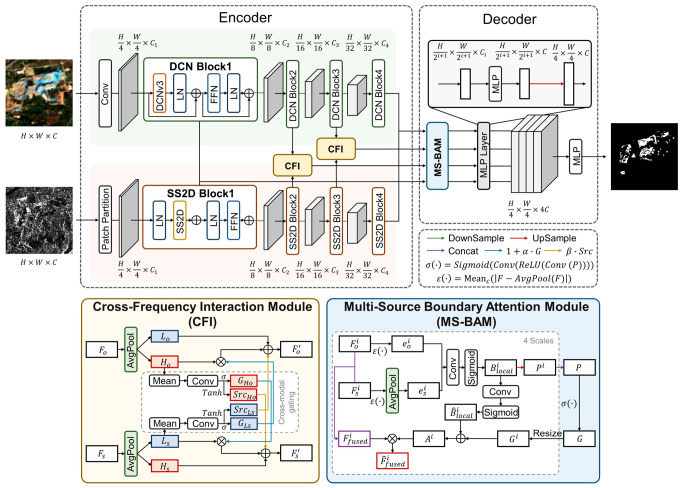
Architecture of the CDW-Net framework.

To address the challenges of irregular target morphologies, fragmented boundaries, and susceptibility to confusion with bare land, construction sites, and hardened surfaces in complex urban backgrounds, this study proposes a dual-branch optical-SAR fusion identification framework named CDW-Net (Figure 4). The framework fully leverages the complementary nature of optical and SAR imagery in land cover representation. Specifically, the optical branch utilizes InternImage to extract color, texture, and local morphological features, while the SAR branch adopts VMamba to model backscattering structures and long-range spatial dependencies. On this basis, a cross-frequency fusion and boundary enhancement mechanism specifically tailored for multi-source remote sensing feature interaction is designed. First, a CFI module is proposed, which decomposes the optical and SAR features into low-frequency and high-frequency components, achieving complementary enhancement of optical boundary details and SAR structural information through asymmetric bidirectional cross-frequency guidance. Second, an MS-BAM is designed to extract local boundary responses from multi-scale features and construct global boundary priors. The locally-globally consistent boundary attention is then re-injected into the fused features to enhance the representation capability of the irregular C&DW boundaries. Finally, a lightweight Segformer decoder is utilized to aggregate the enhanced features across four scales and output the final identification results. Its MLP-based structure projects the heterogeneous multi-scale features into a unified representation space, enabling efficient cross-scale fusion with limited computational overhead. Through the synergistic effects of dual-branch feature learning, cross-frequency interaction, and multi-scale boundary correction, the proposed method effectively enhances the separability between C&DW and complex background features, ultimately improving both the identification accuracy and boundary integrity of C&DW in complex urban environments.

#### 3.2.1. Cross-Frequency Interaction (CFI) Module

To deeply mine the complementary relationship between optical and SAR features at the frequency band level, this study designs the CFI. Unlike direct concatenation or channel-weighted fusion, CFI focuses on the differentiated expressions of the two modalities within low-frequency structural information and high-frequency detailed information. In optical features, high-frequency responses typically correspond to target edges, abrupt texture changes, and local morphological variations; conversely, low-frequency responses in SAR features more readily reflect stable regional structures and background scattering patterns. Therefore, this study models cross-modal fusion as a directional interaction process between frequency bands, leveraging optical high-frequency information to guide the SAR structural response while utilizing SAR low-frequency information to constrain the optical detailed expression.

The optical and SAR features at the i-th scale are denoted as $F_{o}^{i}$ and $F_{s}^{i}$, respectively. The CFI primarily operates on the intermediate semantic scales to introduce robust semantic expression capability while preserving spatial details. The specific procedure is as follows:

First, local average pooling with a 3 × 3 kernel, a stride of 1, and a padding of 1 is applied to both the optical and SAR features to obtain their respective low-frequency components. Subsequently, the low-frequency components are subtracted from the original features to extract the corresponding high-frequency components:

(2) $$L_{o}^{i}=AvgPool\left(F_{o}^{i}\right),\;\;H_{o}^{i}=F_{o}^{i}-L_{o}^{i}$$

(3) $$L_{s}^{i}=AvgPool\left(F_{s}^{i}\right),\;\;H_{s}^{i}=F_{s}^{i}-L_{s}^{i}$$

where $L_{o}^{i}$ and $L_{s}^{i}$ denote the low-frequency components of the optical and SAR features, respectively, which primarily correspond to smoother structures and background information. $H_{o}^{i}$ and $H_{s}^{i}$ represent the high-frequency components of the optical and SAR features, respectively, primarily corresponding to local edges, texture variations, and detailed responses.

Subsequently, the optical high-frequency component and the SAR low-frequency component are utilized to construct raw gating maps for two distinct directions. Specifically, the optical-to-SAR direction is generated from $H_{o}^{i}$ to guide boundaries and texture details, whereas the SAR-to-optical direction is generated from $L_{s}^{i}$ to provide a more stable structural constraint:

(4) $$G_{o-s}^{i,\;raw}=Mean_{c}\left(H_{o}^{i}\right)$$

(5) $$G_{s-o}^{i,raw}=Mean_{c}\left(L_{s}^{i}\right)$$

On this basis, spatial convolution and the Sigmoid function are applied to refine the raw gating maps, yielding the gating weights for both directions. Simultaneously, the Tanh function is utilized to construct the corresponding additive cross-frequency source terms:

(6) $$G_{HO}^{i}=\sigma \left(Conv\left(G_{o-s}^{i,\;raw}\right)\right),\;\;Src_{HO}^{i}=\tanh\left(G_{o-s}^{i,\;raw}\right)$$

(7) $$G_{LS}^{i}=\sigma \left(Conv\left(G_{s-o}^{i,raw}\right)\right),\;\;Src_{LS}^{i}=\tanh\left(G_{s-o}^{i,raw}\right)$$

where $Mean_{c}\left(\cdot \right)$ denotes the average operation along the channel dimension. $G_{o-s}^{i,raw}$ and $G_{s-o}^{i,raw}$ represent the raw gating maps for the two directions, respectively. $G_{HO}^{i}$ and $G_{LS}^{i}$ represent the gating weights refined by spatial convolution. $Src_{HO}^{i}$ and $Src_{LS}^{i}$ are the cross-frequency source terms used to provide additive compensation in addition to multiplicative gating. $\sigma \left(\cdot \right)$ denotes the Sigmoid function. The convolution parameters for the two directions are mutually independent to characterize the asymmetric interaction between the optical-to-SAR and SAR-to-optical pathways.

Finally, the gating weights and cross-frequency source terms are utilized to update the SAR low-frequency component and the optical high-frequency component, respectively. Specifically, the optical-to-SAR pathway modulates $L_{s}^{i}$, while the SAR-to-optical pathway modulates $H_{o}^{i}$:

(8) $${\hat{L}}_{s}^{i}=L_{s}^{i}⊙\left(1+\alpha_{o-s}G_{HO}^{i}\right)+\beta_{o-s}Src_{HO}^{i}$$

(9) $${\hat{H}}_{o}^{i}=H_{o}^{i}⊙\left(1+\alpha_{s-o}G_{LS}^{i}\right)+\beta_{s-o}Src_{LS}^{i}$$

Subsequently, the updated frequency components are recombined with the unreplaced original components:

(10) $$F_{o}^{i,\prime }=L_{o}^{i}+{\hat{H}}_{o}^{i},\;\;F_{s}^{i,\prime }={\hat{L}}_{s}^{i}+H_{s}^{i}$$

where ${\hat{L}}_{s}^{i}$ represents the SAR low-frequency component guided by the optical high frequency, and ${\hat{H}}_{o}^{i}$ represents the optical high-frequency component guided by the SAR low frequency. $\alpha_{o-s}$ and $\alpha_{s-o}$ denote the learnable multiplicative modulation coefficients for the two directions, while $\beta_{o-s}$ and $\beta_{s-o}$ denote the learnable additive compensation coefficients for the two directions. $F_{o}^{i,\prime }$ and $F_{s}^{i,\prime }$ are the enhanced optical and SAR features after CFI processing, respectively. Through directionally independent gating and modulation parameters, the CFI achieves directional and complementary enhancement of optical details and SAR structures without disrupting the original modality representations.

#### 3.2.2. Multi-Source Boundary Attention Module (MS-BAM)

For targets like C&DW that feature fragmented boundaries, irregular morphologies, and significant scale variations, maintaining fine representation within boundary zones presents a distinct challenge. On one hand, optical imagery provides relatively clear texture transitions and edge responses, yet it remains prone to generating pseudo-boundaries within shadows, bare soil, and active construction backgrounds. On the other hand, SAR imagery captures the structural disparities between targets and their surroundings, but its inherent speckle noise can lead to discontinuous boundary responses. Consequently, it is essential to further extract boundary clues from multi-source features and establish consistent boundary constraints across different scales.

To address this, this study designs the MS-BAM. The core philosophy of this module is as follows. First, local boundary responses are extracted from both optical and SAR features at each scale and fused to form intra-scale boundary priors. Subsequently, the boundary maps from different scales are aligned to a unified reference scale to construct a cross-scale global boundary prior. Finally, both the local and global boundary information are co-injected back into the fused features, thereby enhancing the model’s perceptual capability toward the irregular boundaries of C&DW.

Denoting the i-th scale optical and SAR features entering MS-BAM as $F_{o}^{i}$ and $F_{s}^{i}$, respectively, their fused feature can be represented as $F^{i}$. The MS-BAM operates across all four feature scales, and its calculation procedure is as follows:

First, local boundary responses are extracted from the optical and SAR features independently. Specifically, edges, abrupt texture changes, and variations in scattering mechanisms are highlighted by calculating the difference between the original features and their locally smoothed counterparts. For local smoothing, a fixed average pooling operator with a 3 × 3 kernel, a stride of 1, and a padding of 1 is applied consistently across all four feature scales. Simultaneously, considering that SAR features are highly susceptible to speckle noise, an additional local smoothing operation is applied to the SAR boundary response using another fixed 3 × 3 average pooling operator with a stride of 1 and a padding of 1:

(11) $$e_{o}^{i}=Mean_{c}\left(\left|F_{o}^{i}-AvgPool\left(F_{o}^{i}\right)\right|\right)$$

(12) $$e_{s}^{i}=AvgPool\left(Mean_{c}\left(\left|F_{s}^{i}-AvgPool\left(F_{s}^{i}\right)\right|\right)\right)$$

where $e_{o}^{i}$ denotes the local boundary response map derived from the optical features at the i-th scale, and $e_{s}^{i}$ represents the smoothed SAR boundary response.

Subsequently, the optical boundary response is fused with the smoothed SAR boundary response to generate the local boundary prior for the current scale:

(13) $$B_{local}^{i}=\sigma \left(Conv\left(Concat\left[e_{o}^{i},e_{s}^{i}\right]\right)\right)$$

To further introduce cross-scale boundary consistency, all local boundary maps are upsampled to a unified reference scale and aggregated via pyramidal fusion to construct the global boundary prior:

(14) $$P^{i}=Resize\left(B_{local}^{i}\right)$$

(15) $$G=\sigma \left(Conv\left(\phi \left(Conv\left(Concat\left(P^{i}\right)\right)\right)\right)\right)$$

where $P^{i}$ represents the i-th local boundary map aligned to the reference scale, G denotes the global boundary prior obtained by fusing the multi-scale boundary information, and $\phi \left(\cdot \right)$ signifies the rectified linear unit activation function.

Finally, the global boundary prior G  is resized back to the i-th scale to yield $G^{i}$. Simultaneously, the local boundary prior $B_{local}^{i}$ is further refined to obtain ${\hat{B}}_{local}^{i}$:

(16) $$G^{i}=Resize\left(G\right)$$

(17) $${\hat{B}}_{local}^{i}=\sigma \left(Conv\left(B_{local}^{i}\right)\right)$$

Subsequently, the local and global boundary priors are combined to generate the final boundary attention map $A^{i}$:

(18) $$A^{i}=clip\left(0.5{\hat{B}}_{local}^{i}+0.5G^{i},0,1\right)$$

Finally, the $A^{i}$ is injected back into the fused features $F_{fused}^{i}$ in a residual enhancement manner, yielding the boundary-enhanced fused features:

(19) $${\tilde{F}}_{fused}^{i}=F_{fused}^{i}⊙\left(1+A^{i}\right)$$

where ${\hat{B}}_{local}^{i}$ denotes the refined local boundary prior, $A^{i}$ represents the final boundary attention map, $clip\left(\cdot \right)$ denotes a clipping function that constrains the attention values within the range of [0, 1], and ${\tilde{F}}_{fused}^{i}$ signifies the fused features enhanced by the MS-BAM.

### 3.3. Accuracy Evaluation Metrics

Since C&DW occupies only a small proportion of the image compared with the background, the dataset exhibits a pronounced class imbalance. Therefore, IoU and F1-score were adopted as the primary evaluation metrics because they provide a more balanced assessment of segmentation performance, while Precision, Recall, Kappa, and Overall Accuracy (OA) were additionally reported for a comprehensive evaluation.

(20) $$IoU=\frac{TP}{TP+FP+FN}$$

(21) $$Precision=\frac{TP}{TP+FP}$$

(22) $$Recall=\frac{TP}{TP+FN}$$

(23) $$F-score=\frac{2\times Precision\times Recall}{Precision+Recall}$$

(24) $$OA=\frac{TP+TN}{TP+TN+FP+FN}$$

(25) $$Kappa=\frac{p_{o}-p_{e}}{1-p_{e}}\;\left\{\begin{array}{c} p_{o}=OA\\ p_{e}=\frac{\left(TP+FP\right)\left(TP+FN\right)+\left(FN+TN\right)\left(FP+TN\right)}{{\left(TP+TN+FP+FN\right)}^{2}} \end{array}\right.$$

## 4. Results

### 4.1. Comparative Experiments

To ensure the fairness and comparability of the experiments, a unified training configuration was adopted for all comparative and ablation studies in this work. The input patch size was set to 256 × 256 pixels, the batch size was 12, and the model was trained for 100 epochs. The AdamW optimizer was adopted with an initial learning rate of 6 × 10^−5^. All experiments were conducted on a Linux system, powered by an NVIDIA RTX 2080 Ti GPU.

To comprehensively validate the effectiveness of the proposed method, we designed two categories of comparative experiments, comprising nine groups in total. The first category utilizes Unet and Segformer as backbone networks, tested under optical-only, SAR-only, and channel-stacked configurations, respectively. This setup aims to investigate the performance upper bound of single-modality data and the efficacy of early input-level fusion. The second category selects three state-of-the-art dual-branch networks specifically designed for joint optical-SAR segmentation: ASANet [29], MCANet [30], and PAD [31]. This category serves to demonstrate the superiority of our proposed fusion framework over existing joint segmentation schemes.

As presented in Table 2, the proposed method achieves the top performance across all six evaluation metrics, yielding an IoU of 0.8470 and an F1-score of 0.9171. Given the pronounced class imbalance between the dominant background pixels and the relatively limited C&DW regions, these two metrics were regarded as the primary indicators of segmentation performance. Compared to the single-modality baselines, our method improves the IoU and F1-score by approximately 8 and 5 percentage points over Unet-optical/SAR, and by more than 14 percentage points for both comprehensive metrics over Segformer-optical/SAR. This confirms the substantial complementarity between optical and SAR data, demonstrating that a single modality struggles to meet the segmentation demands of complex scenarios. Compared to early-stage channel-stacking fusion, our method outperforms Unet-stack by 6.22 and 3.77 percentage points in IoU and F1-score, respectively, with an even more pronounced improvement over Segformer-stack. This proves that simple input-level concatenation fails to fully exploit the complementary relationships of heterogeneous data, underscoring the necessity of more refined interaction modeling at the feature level. Furthermore, compared to the dual-branch multimodal networks, our method improves the IoU and F1-score by 3.33 and 1.98 percentage points, respectively, and boosts Precision and Kappa by 2.72 and 2.14 percentage points over MCANet, which is the top-performing baseline. The advantages are even more striking when compared against ASANet and PAD, where our model exhibits an IoU improvement exceeding 15 percentage points and an F1-score gain of nearly 10 to 15 percentage points. Notably, our method achieves the highest Precision (0.9145) among all compared models while maintaining a high Recall (0.9198), demonstrating a well-balanced performance between precision and recall. These results fully substantiate the effectiveness and state-of-the-art performance of the proposed joint optical-SAR segmentation framework in suppressing false alarms while preserving authentic targets.

**Figure 5:**
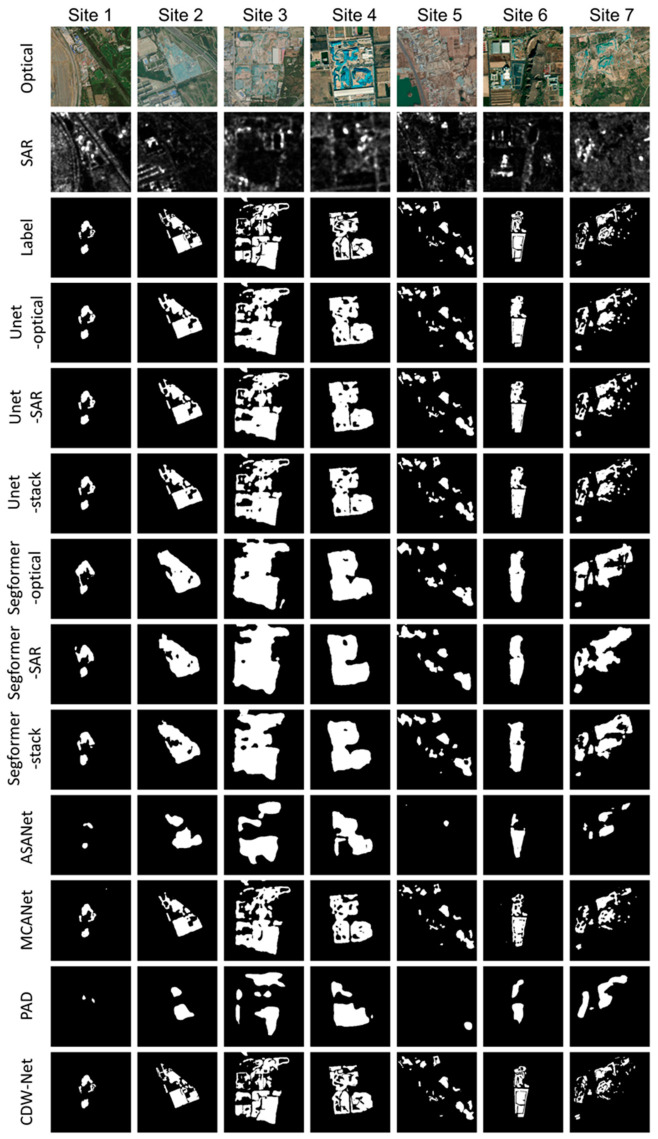
Visual comparison of identification results from different methods.

To further evaluate the identification performance of each method across various scenarios, seven representative typical regions were selected for refined comparative analysis (Figure 5). Site 1 through Site 3 are located within urban built-up areas or inner suburbs. Among them, Site 1 and Site 2 feature relatively simple backgrounds with clear target boundaries, whereas Site 3 presents an increased identification difficulty due to its large target area and irregular boundaries. Site 4 and Site 5 are situated in suburban or plain agricultural areas, where croplands and buildings interweave dynamically. Notably, the target distribution in Site 5 is exceptionally scattered and fragmented, introducing significant background interference. Site 6 and Site 7 are positioned in near-mountain areas or mountain fringes; although the targets there are small and relatively regular in shape, the recognition difficulty remains high due to the mountain topography and complex land-cover backgrounds. Encompassing diverse typical scenarios such as urban, suburban, agricultural, and mountainous environments, these seven regions comprehensively reflect the generalization capability of the algorithms under varying levels of environmental complexity.

From the visualization results of these typical regions, the proposed method exhibits distinct advantages across all scenarios. In the urban and suburban contexts of Site 1 to Site 3, compared with Unet, Segformer, and MCANet, the proposed method recovers more internal details and edge structures of the targets. When compared against ASANet and PAD, our identification results are noticeably more complete with a marked reduction in missed detections, achieving high classification accuracy in both regular and irregular target shape scenarios. In the mixed agricultural scenarios of Site 4 and Site 5, the proposed method also substantially outperforms the other competing models. Especially in the complex case of Site 5, where targets are extremely scattered, and background interference is severe, the recognition results of ASANet and PAD fail almost completely, whereas our method still extracts the target areas quite accurately, demonstrating superior robustness. In the near-mountain scenarios of Site 6 and Site 7, the proposed method likewise achieves the optimal classification results, surpassing other approaches in both small-target identification and complex background suppression. This further validates the effectiveness and generalization capability of the proposed method across diverse and challenging scenarios.

### 4.2. Ablation Study

To verify the effectiveness of each module design and the individual contributions of the dual-modality data, this study conducted six groups of ablation experiments: the Baseline represents the fundamental backbone network; Baseline + CFI and Baseline + MS-BAM are utilized to evaluate the standalone performance of each module; CDW-Net (optical) and CDW-Net (SAR) serve to assess the contribution of each respective modality; and CDW-Net constitutes the complete model integrating all proposed modules and dual-modality inputs. All experiments were conducted under identical training parameters, and a comprehensive evaluation was performed using six metrics: IoU, Precision, F1-score, Recall, Kappa, and OA (Table 3).

From the module perspective, compared to the Baseline (IoU = 0.8006), the integration of the CFI module increases the IoU to 0.8207, demonstrating that this module effectively alleviates the discrepancies between heterogeneous features to achieve complementary cross-modal fusion. The incorporation of the MS-BAM module boosts the IoU to 0.8241 and delivers a Precision of 0.9007, which highlights its distinct advantages in multi-scale feature aggregation and boundary delineation. When both modules operate synergistically, the complete model achieves an IoU, F1-score, and Kappa of 0.8470, 0.9171, and 0.9114, respectively—marking improvements of 4.64, 2.78, and 2.99 percentage points over the Baseline. This validates their mutual complementarity and the rationality of the overall architecture design.

From the modality perspective, using optical data alone yields an IoU of only 0.7803, as it is noticeably constrained by cloud cover and illumination conditions. Utilizing SAR data alone achieves an IoU of 0.7965, exhibiting better overall robustness, yet it still suffers from accuracy loss due to inherent speckle noise. Upon fusing both modalities, the IoU improves by 6.67 and 5.05 percentage points compared to the optical-only and SAR-only configurations, respectively. This demonstrates that the spectral-textural information from optical imagery and the geometric-structural information from SAR imagery are highly complementary, effectively overcoming the limitations of single-source data to deliver more precise and stable identification results.

To evaluate the selection of encoders for each modality, we conducted ablation studies on both the OPT-Encoder and SAR-Encoder. Specifically, we fixed the SAR-Encoder as VMamba to evaluate different OPT-Encoder architectures, and subsequently fixed the OPT-Encoder as InternImage to assess alternative SAR-Encoder structures. The results are summarized in Table 4. In the OPT-Encoder comparison, ResNet50 and MixVisionTransformer (Mit) yielded IoU values of 0.8108 and 0.8204, respectively, both falling short of InternImage’s 0.8470. This demonstrates that the deformable convolution mechanism of InternImage is better suited for feature extraction from optical imagery. In the SAR-Encoder comparison, VMamba consistently outperformed ResNet50 (0.8111) and Mit (0.7937), confirming that its state space model is more effective at modeling the long-range dependencies inherent in SAR scattering textures. In conclusion, the finalized combination of InternImage and VMamba adopted in this study achieved the optimal performance across all configuration pairings.

### 4.3. Model Transfer Application

**Figure 6:**
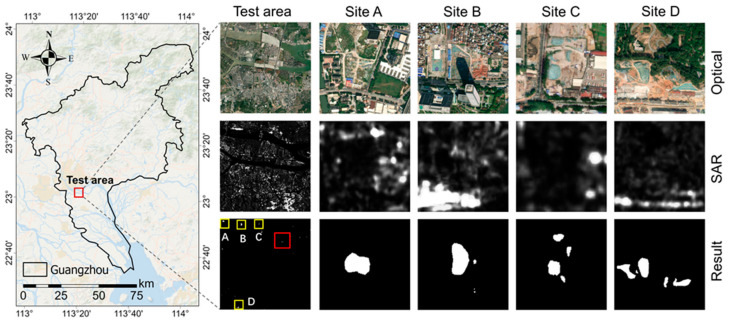
Generalizability validation of the model (taking the Guangzhou site as an example).

To verify the generalizability of the proposed model, an independent area covering approximately 6000 hectares in Guangzhou was selected for a C&DW detection experiment. Located within an urban built-up area, this region encompasses a variety of complex land covers, including buildings, roads, bare land, and construction sites. The heavy background interference poses a high recognition challenge. Based on data availability, optical and SAR remote sensing data acquired on 17 July 2023, were utilized for CDW identification, and the detection results are illustrated in Figure 6.

The results indicate that the model can accurately extract the spatial distribution of C&DW, successfully identifying four distinct C&DW dumping sites within the study area, which demonstrates the robust cross-regional generalization capability of the proposed method. Based on manually interpreted validation samples, the proposed method achieved an IoU of 0.8247 and an F1-score of 0.9040, further demonstrating its cross-regional transferability. Specifically, Site A is characterized by mixed surrounding land-cover types, while Site B is in a densely built-up urban area. The complex backgrounds in these two areas lead to some missed detections. In contrast, Site C is adjacent to bare land, and Site D is mainly surrounded by vegetation, where the model produces more complete extraction results. These observations further demonstrate the adaptability of the proposed optical-SAR fusion framework to different surrounding environments. The performance of CDW-Net across these heterogeneous scenes benefits from the complementary interaction between optical details and SAR structural information provided by the CFI module, together with the boundary refinement of MS-BAM. Compared with single-modal or simple fusion approaches, this design enhances background discrimination and boundary preservation under complex urban conditions. Concurrently, a minor degree of misclassification persists, as indicated by the red boxes in Figure 6, where certain building areas were incorrectly identified as C&DW. The primary reason for this is that C&DW shares spectral and SAR backscattering similarities with some buildings; both exhibit strong texture variations and high backscattering characteristics, leading to localized confusion. Overall, these misclassified areas are limited and do not affect the model’s ability to capture the primary C&DW regions.

### 4.4. Model Performance Analysis

To comprehensively evaluate the efficiency of the proposed CDW-Net, we systematically compared it with three representative dual-branch optical-SAR fusion methods (ASANet, MCANet, and PAD) in terms of parameter size, computational complexity, and inference speed. The results are summarized in Table 5. Latency and FPS were evaluated using 256 × 256 input patches, and the reported values represent the average performance over repeated inference runs. Benefiting from the compact feature representations of the InternImage and VMamba dual branches, as well as the decoupling of cross-modal interactions within the frequency domain by the CFI module, CDW-Net completes inference with only 59.12 M parameters and 14.11 GFLOPs. Both its parameter count and computational complexity are the lowest among the four methods, marking reductions of 16.41% and 80.20%, respectively, compared to the second-best method, MCANet. Regarding inference speed, CDW-Net achieves 4.0482 FPS with a single-image latency of 0.2470 s, exhibiting a small standard deviation across multiple tests and demonstrating excellent operational stability. Overall, CDW-Net achieves the optimal identification accuracy (IoU = 0.8470) with the smallest model scale, lowest computational overhead, and fastest inference speed. It strikes a favorable balance between model capacity and computational cost, demonstrating strong deployment potential for large-scale C&DW identification tasks in complex urban environments.

## 5. Discussion

### 5.1. Architectural Advantages of CDW-Net

Existing C&DW extraction studies have mainly relied on single-source optical imagery and have improved feature representation through attention mechanisms, or multi-band fusion [19,21,22]. For example, CCMGNet employs dual RGB-NIR encoders with gated multi-scale fusion, while CBAM-U-Net enhances feature extraction through channel and spatial attention mechanisms. CCMGNet reported a Precision of 0.8942 and an IoU of 0.8452, whereas other CNN-based studies reported markedly different performance depending on the target type, imagery, and experimental setting. These results provide a useful context for evaluating recent C&DW extraction methods, although their main information sources remain optical spectral and textural features, with limited use of structural differences in complex urban environments. In contrast, the proposed method constructs a hybrid framework based on the complementary characteristics of optical and SAR data. Optical imagery provides rich color, texture, and local morphological information, whereas SAR imagery contributes complementary structural, surface roughness, and backscattering characteristics. To effectively exploit these complementary features, CDW-Net adopts a CNN-Mamba heterogeneous dual-branch encoder with a Transformer decoder, where the optical branch focuses on local detail extraction, the SAR branch models long-range structural dependencies, and the Transformer decoder performs multi-scale semantic aggregation. Furthermore, the proposed CFI module explicitly facilitates the interaction between optical high-frequency details and SAR low-frequency structural information, while the MS-BAM enhances the delineation of fragmented and irregular C&DW boundaries through both local and global boundary constraints. Compared with conventional CNN-based methods, which mainly emphasize local feature extraction, and Transformer-based models, which primarily focus on global contextual modeling, the proposed hybrid architecture effectively integrates local texture representation, long-range dependency modeling, multimodal feature complementarity, and fine boundary delineation.

Under the unified experimental setting, CDW-Net achieved an F1-score of 0.9171, a Recall of 0.9198, and a Precision of 0.9145. The simultaneously high Recall and Precision indicate that the model effectively reduces missed detections while suppressing false positives in complex urban backgrounds. Combined with the consistent improvements observed in the ablation experiments after introducing the CFI and MS-BAM modules, these results suggest that the superior performance primarily stems from the complementary utilization of optical and SAR information, long-range structural modeling, and enhanced boundary representation, rather than simply increasing model complexity. Overall, the performance improvement of CDW-Net can be attributed to the effective integration of complementary multimodal information and the joint modeling of long-range structural dependencies and irregular target boundaries.

### 5.2. Limitations and Future Prospects

Although this study constructs the first optical-SAR multimodal semantic segmentation dataset tailored for the refined extraction of C&DW and proposes the CDW-Net fusion framework to achieve superior identification accuracy across various complex urban scenarios, certain limitations remain. First, the samples in the current dataset are primarily collected from the Beijing area, offering limited geographical diversity. Although the transfer experiment in Guangzhou demonstrates promising cross-regional applicability, the generalization capability of CDW-Net across more diverse geographic regions and different optical-SAR sensor combinations still requires further validation. Future work will therefore expand the dataset to additional regions and sensor configurations. Second, the SAR data utilized in this study primarily consist of dual-polarization observations. Future research could introduce quad-polarization SAR data and incorporate polarimetric decomposition methods to conduct a deeper physical analysis of the scattering mechanisms of C&DW, thereby further strengthening the model’s discriminative capability for complex land covers. In addition, future studies will investigate the incorporation of additional Sentinel-2 spectral bands, such as the Near-Infrared (NIR) and Short-Wave Infrared (SWIR) bands, into the proposed optical-SAR framework to further improve the discrimination of C&DW from spectrally similar land-cover types. Moreover, higher resolution multi-source remote sensing data will be explored in future studies to further improve the representation of fragmented C&DW boundaries. Furthermore, this study focuses on the static identification of C&DW based on single-temporal imagery, without addressing the monitoring demands of its dynamic variations across construction life cycles. Future efforts will explore the integration of multi-temporal remote sensing observations to support dynamic change analysis and long-term sequence mapping of C&DW spatial distributions.

## 6. Conclusions

To address the challenges posed by scattered C&DW dumping sites, complex background interference, and fragmented boundaries in urban environments, this study proposes a multimodal remote sensing framework for fine-grained C&DW extraction. First, to overcome the limitation of existing datasets that rely on single-source data and cannot adequately support pixel-level extraction tasks, we construct the first optical-SAR multimodal dataset for C&DW semantic segmentation, filling the gap in multimodal collaborative monitoring data. Furthermore, to integrate the spectral information of optical imagery with the structural information of SAR imagery while addressing the irregular boundaries of C&DW areas, we propose CDW-Net, a dual-branch optical-SAR fusion framework. Built upon a CNN-Mamba heterogeneous encoder and a Transformer-based decoder, the framework enables effective modeling of local detailed features and long-range spatial dependencies, highlighting the potential of heterogeneous architectures for multimodal remote sensing interpretation. To facilitate interaction between heterogeneous modalities, the CFI module is introduced to achieve complementary enhancement of optical high-frequency details and SAR structural information through asymmetric guidance. In addition, the MS-BAM is designed to improve fragmented boundary representation in complex backgrounds by integrating local boundary responses with global boundary priors. Experimental results demonstrate that the proposed framework effectively improves C&DW extraction performance across diverse scenarios in Beijing, including urban, suburban, agricultural, and mountainous areas. CDW-Net consistently outperforms single-modal baselines and existing multimodal segmentation methods across all evaluation metrics, achieving superior segmentation performance over representative state-of-the-art methods. Moreover, the ablation study verifies the effectiveness of the proposed CFI and MS-BAM modules. Transfer experiments conducted in Guangzhou further demonstrate the strong cross-regional generalization capability of the proposed framework. Overall, this study provides an effective technical framework for large-scale remote sensing monitoring of urban C&DW.

## Figures and Tables

**Table 1:** Feature combinations for C&DW identification.

Feature Group	Feature Name	Description
SAR	HH	HH polarization backscatter intensity
	VH	VH polarization backscatter intensity
	${\mathrm{CV}}_{HH}$	Coefficient of variation of HH backscatter
Optical	R	Red band reflectance
	G	Green band reflectance
	B	Blue band reflectance

**Table 2:** Accuracy metrics of comparative experiments.

Model	IoU	Precision	F1-Score	Recall	Kappa	OA
Unet—optical	0.7667	0.8458	0.8680	0.8913	0.8583	0.9820
Unet—SAR	0.7667	0.8376	0.8679	0.9005	0.8585	0.9824
Unet—stack	0.7848	0.8660	0.8794	0.8932	0.8707	0.9837
Segformer—optical	0.6264	0.6793	0.7703	0.8894	0.7520	0.9655
Segformer—SAR	0.6047	0.6611	0.7537	0.8763	0.7331	0.9613
Segformer—stack	0.6398	0.7032	0.7804	0.8765	0.7633	0.9679
ASANet	0.6947	0.8179	0.8198	0.8218	0.8073	0.9765
MCANet	0.8137	0.8873	0.8973	0.9075	0.8900	0.9865
PAD	0.6283	0.7695	0.7717	0.7739	0.7558	0.9703
CDW-Net (Ours)	0.8470	0.9145	0.9171	0.9198	0.9114	0.9892

**Table 3:** Accuracy metrics of module and modality ablation experiments.

Model	IoU	Precision	F1-Score	Recall	Kappa	OA
Baseline	0.8006	0.8790	0.8893	0.8997	0.8814	0.9854
Baseline + CFI	0.8207	0.8971	0.9015	0.9060	0.8946	0.9871
Baseline + MS-BAM	0.8241	0.9007	0.9036	0.9064	0.8968	0.9874
CDW-Net (optical)	0.7803	0.8734	0.8766	0.8798	0.8680	0.9839
CDW-Net (SAR)	0.7965	0.8742	0.8867	0.8995	0.8787	0.9851
CDW-Net	0.8470	0.9145	0.9171	0.9198	0.9114	0.9892

**Table 4:** Ablation analysis of different encoder structures.

OPT-Encoder	SAR-Encoder	IoU	Precision	F1-Score	Recall	Kappa	OA
InternImage	ResNet50	0.8111	0.8910	0.8957	0.9005	0.8884	0.9864
InternImage	Mit	0.7937	0.8780	0.8850	0.8921	0.8769	0.9849
ResNet50	VMamba	0.8108	0.8875	0.8955	0.9036	0.8882	0.9864
Mit	VMamba	0.8204	0.8972	0.9014	0.9056	0.8945	0.9871
InternImage	VMamba	0.8470	0.9145	0.9171	0.9198	0.9114	0.9892

**Table 5:** Model efficiency comparison.

Model	IoU	Params (M)	GFLOPs	Latency (s/img)	FPS
ASANet	0.6947	82.90	25.92	0.2494 ± 0.0023	4.0105 ± 0.0361
MCANet	0.8137	70.73	71.26	0.2472 ± 0.0013	4.0449 ± 0.0205
PAD	0.6283	74.13	26.20	0.2572 ± 0.0070	3.8895 ± 0.1065
CDW-Net	0.8470	59.12	14.11	0.2470 ± 0.0029	4.0482 ± 0.0337

## Data Availability

The original data presented in this study are openly available in GitHub at https://github.com/charlieee-SAR/CDWNet-v1 (accessed on 17 July 2026). The experiments were conducted using Python 3.10, PyTorch 2.1.2.
